# Impact of visual impairment on physical activity in early and late age-related macular degeneration

**DOI:** 10.1371/journal.pone.0222045

**Published:** 2019-10-21

**Authors:** Manuel Heinemann, Susanne G. Welker, Jeany Q. Li, Maximilian W. M. Wintergerst, Gabrielle N. Turski, Christopher A. Turski, Jan H. Terheyden, Matthias M. Mauschitz, Frank G. Holz, Robert P. Finger

**Affiliations:** University of Bonn, Department for Ophthalmology, Ernst- Abbe- Straße, Bonn, Germany; National Yang-Ming University Hospital, TAIWAN

## Abstract

**Background:**

Modifiable risk factors for age-related macular degeneration (AMD) include smoking, nutrition and likely physical activity (PA). Levels of PA, however, are impacted by any visual impairment which makes the assessment of any association with AMD difficult.

**Purpose:**

To assess the impact of visual impairment under both high and low luminance conditions on levels of PA in early and late AMD.

**Methods:**

Ninety participants with early to late AMD underwent a clinical assessment including conventional best-corrected visual acuity, low luminance visual acuity, contrast sensitivity and the Moorfields acuity test. PA was recorded using a wrist-worn accelerometer (GENEActiv, Activeinsights) on seven consecutive days. Patient characteristics were compared with the Wilcoxon rank-sum test and determinants of moderate-to-vigorous-PA (MVPA) were assessed using linear regression models.

**Results:**

Mean age was 73.9 ± 8.5 years (range 50–89) and 47 subjects (52.2%) were women. Average MVPA time was longer in the early (355.1 ± 252.0 minutes/week) compared to the late AMD group (162.2 ± 134.6 minutes/week; p<0.001). Using linear regression, age [β = -0.25; 95% confidence interval (CI): -12.9; -0.8, p = 0.028] and AMD stage (β = -0.28; 95% CI: -230.9, -25.0; p = 0.015) but not visual impairment on any of the employed tests were associated with MVPA (minutes/week).

**Conclusions:**

We found late AMD to be associated with reduced PA. As performance on any of the visual tests was not associated with PA, this association cannot entirely be explained by functional impairment. More research is needed to further explore the association of PA and AMD as PA may be a potentially modifiable risk factor.

## Introduction

Age-related macular degeneration (AMD) is the main cause of severe visual impairment and blindness in industrialized countries. [1,2] AMD can be classified into early, intermediate and late stage disease. [3] While patients with early and intermediate AMD tend to have no or only little visual impairment, late stage AMD can severely reduce visual acuity and, thus, impact quality of life. [4,5] Known risk factors include age, genetic predisposition, smoking, nutrition and likely physical activity (PA). [6–15]

Moderate to high levels of PA have been described as a protective factor in various diseases [16–21], even if PA is increased only later in life. [22] A systematic review and meta-analysis by McGuiness and colleagues found lower odds of early and late AMD in those with above average levels of PA [11] The *Blue Mountains Eye Study* reported lower levels of PA in participants with late AMD compared to early, but did not find PA to lower the risk of incident AMD. [6] Conversely, the Beaver Dam Eye Study reported high levels of PA to be protective for incident visual impairment irrespective of underlying pathology. [7] Thus, the status of PA as an AMD risk factor today is controversial and confounded by the visual impairment occurring concurrently with AMD progression. [11,23,24] Most of the studies to date have quantified PA using self-report, i.e. questionnaires, with very few studies employing objective measurements based on e.g. accelerometers (ACCs). [9,25,26]

Herein, we assessed any visual impairment under high and low luminance as well as levels of PA using an accelerometer in persons with various stages of AMD in order to better characterize the relationship of PA, visual impairment and AMD.

## Materials and methods

### Participants

We enrolled 90 participants aged 50 years and above with a diagnosis of AMD. All patients underwent a dilated fundus exam by an ophthalmologist and multi-modal retinal imaging including infrared reflectance, spectral domain—optical coherence tomography and fluorescein angiography if deemed necessary by the assessing physician (SD-OCT; Spectralis, Heidelberg Engineering). Maximum drusen size and signs of late AMD (atrophy or signs associated with CNV) were determined based on this multi-modal approach. AMD classification was based on the worse eye in accordance with the recently published clinical classification of AMD. [3] Participants were categorized into “early AMD” (including participants with early and intermediate AMD) and “late AMD” based on the more advanced eye and in accordance to the *Beckman Initiative for Macular Research Classification Committee*. [3]

All patients underwent a standardized interview on demographic factors, medical and ophthalmic history and awareness of eye diseases. Subsequently, participants were clinically assessed and underwent visual function tests. After this, they were equipped with the wrist-worn ACCs. Assessments took place between January 2017 and January 2019 at the Department of Ophthalmology at the University of Bonn, Germany.

The study was approved by the Institutional Review Board of the University of Bonn, Germany (approval ID: 013/16). Written informed consent was obtained from all participants following the guidelines of the Declaration of Helsinki and the International Conference on Harmonization of Good Clinical Practice (ICH-GCP).

### Physical activity measurement

Everyday PA was assessed using the GENEActiv ACC (Activeinsights, Kimbolton, United Kingdom). Participants are required to wear the ACC continuously (i.e. for 24 hours a day) on seven consecutive days including the weekend on their non-dominant wrist. [27] As the devices are waterproof, they do not need to be taken off for any activities involving contact with water. The GENEActiv ACC is based on a microelectronic- mechanic system, which documents acceleration in *g* in all of the three dimensions (1g = 9,81 m/s^2^; max. possible acceleration: 8*g* ± 3,9*mg*). Frequency of data collection for the seven days was 100 Hz. [28,29] We summarized PA data into minutes per week for sedentary, low, moderate, vigorous and moderate-to-vigorous (MVPA) activity. [27]

### Visual function tests

All participants underwent the following best-corrected visual function tests: high-luminance visual acuity (i.e. conventional VA, HLVA), low luminance visual acuity (LLVA) as well as the Moorfields acuity test (MAT) at a standard distance of four meters and recorded the maximum letters. If the patient was unable to read the first four rows of the test, the distance was reduced to one meter. [30] For HLVA and LLVA, Early Treatment Diabetic Retinopathy Study (ETDRS) charts were used in a standard retro-illumination box. After testing HLVA, an additional 2log-unit density filter was placed in the trial frame. The MAT follows the same procedure as HLVA, with a modified test chart based on the ETDRS chart with an additional high pass filter, to simulate lower contrast situations. [31] Subsequently, we performed the Pelli-Robson contrast sensitivity test at a distance of one meter. [32]

### Data analysis

Data were recorded electronically using Excel (Version 14.0, Microsoft, Washington, USA). Groups were compared using Wilcoxon rank-sum tests for continuous variables and Mann-Whitney test for categorical variables. Determinants of MVPA were assessed using linear regression models. Analyses were performed with STATA 13 (StataCorp, College Station, USA). P-values below 0.05 were considered statistically significant.

## Results

Mean (SD) age of the 90 participants was 73.9 ± 8.5 years (range 50–89) and 47 subjects were women. Patients with early AMD were on average younger than those with late neovascular AMD and had a higher MVPA (p<0.001, Table 1). BMI was not different between early and late AMD (p = 0.23).

**Table 1:** Participant characteristics.

Characteristics	n (%) or mean ± SD			
	all	early AMD	late AMD	p-value
n (%)	90 (100%)	49 (54.4%)	41 (45.6%)	
Age (in years)	73.9 ± 8.5	69.6 ± 7.8	79.1 ± 6.0	**<0.001**
Range (in years)	50–89	50–84	63–89	
Sex Male	43 (47.8%)	17 (34.7%)	16 (39%)	0.67
Female	47 (52.2%)	32 (65.3%)	25 (61%)	
BMI (kg/m^2^)		25.6 ± 3.3	26.8 ± 4.7	0.23
MVPA (min/week)		355.1 ± 252.0	162.2 ± 134.6	**<0.001**

SD = standard deviation BMI = body mass index; MVPA = moderate-to-vigorous-activity (minutes per week)

### Visual functional tests

Participants in the early AMD group, overall performed better on all functional tests compared with the late AMD group (p<0.001; Table 2).

**Table 2:** Performance on visual function tests.

	Statistic	early AMD	late AMD	p- value *
HLVA	Mean ± SD	82.8 ± 6.9	54.2 ± 15.9	**<0.001**
	Min; Median; Max	62 ; 84 ; 95	12 ; 58 ; 79	
LLVA	Mean ± SD	65.9 ± 9.6	35.4 ± 16.0	**<0.001**
	Min; Median; Max	40 ; 68 ; 85	0 ; 38 ; 68	
MAT	Mean ± SD	61.1 ± 6.9	38.9 ± 12.6	**<0.001**
	Min; Median; Max	48 ; 62 ; 79	0 ; 42 ; 60	
Pelli-Robson	Mean ± SD	34.1 ± 3.6	24.8 ± 7.6	**<0.001**
	Min; Median; Max	20 ; 34 ; 42	0 ; 28 ; 35	

*Wilcoxon rank-sum test; SD = standard deviation; HLVA = high luminance best-corrected visual acuitiy, LLVA = low-luminance visual acuity, MAT = Moorfields Vanishing Optotypes Acuity Test, Pelli-Robson = Pelli Robson contrast sensitivity

### Determinants of PA

Assessing determinants of MVPA irrespective of any AMD classification, both age (β = -0.28; 95% CI: -13.3; -1.7, p = 0.012) and LLVA (β = 0.27; 95% CI: 0.6; 5.5, p = 0.016) were independently associated (Table 3). Including AMD status into the model, we found age (β = -0.25; 95% CI: -12.9; -0.8, p = 0.028) and AMD stage (β = -0.28; 95% CI: -230.9; -25.0, p = 0.015) to be determinants of MVPA, while performance on none of the functional tests remained associated (Table 4). Assessing determinants of MVPA in early and late AMD separately, HLVA was associated with MVPA in the early AMD group only (OR = 1.45; 95% CI 3.34 ; 23.45).

**Table 3:** Determinants of MVPA irrespective of AMD status.

	Beta coefficient	95% Conf. Interval		p- value*
Age	-0.28	-13.3	-1.7	0.012
LLVA	0.27	0.6	5.5	0.016

* Multivariate linear regression model, MVPA = moderate-to-vigorous physical activity

AMD = Age-related macular degeneration, LLVA = low-luminance visual acuity

**Table 4:** Determinants of MVPA including AMD status.

	Beta coefficient	95% Conf. Interval		p- value*
Age	-0.25	-12.9	-0.8	**0.028**
AMD stage	-0.28	-230.9	-25.0	**0.015**

* Multivariate linear regression model, MVPA = moderate-to-vigorous physical activity

AMD = Age-related macular degeneration

## Discussion

In this study we found age and stage of AMD to be determinants of MVPA, which was much lower in participants with late AMD. LLVA was the only functional outcome associated with MVPA across the whole sample, but no functional tests remained associated after controlling for AMD stage and age. Thus, the association of AMD stage and MVPA cannot entirely be explained by functional loss. More research is needed to clarify the association of PA and AMD as PA may be a potentially modifiable AMD risk factor.

Higher levels of vigorous exercise have been reported to be associated with reduced incident risk for any AMD [33] and early AMD. [11] In cross-sectional studies, late AMD was consistently associated with lower levels of PA. [9,34] However, study comparisons are limited as most studies have used different questionnaires[6,8,10,24,35,36] or one- to three-dimensional non-waterproof ACCs [9,25,26,34] to quantify PA. Questionnaires have been shown to over-estimate PA, with objective means to quantify PA such as accelerometers yielding lower levels of PA compared to questionnaires. [28] Non-waterproof ACCs need to be taken off for any activity involving contact with water, which likely includes any household cleaning activity, so is likely to considerably underestimate PA. [37] Taking this into consideration, our results using a waterproof, wrist-worn ACCs are likely to be representative of actual levels of PA. Overall results are in keeping with other published studies, reporting lower levels of PA in late AMD compared to earlier stages of the disease and controls. [9,26,34]

Compared to previous studies, which used a number of existing or self-developed AMD classification systems [6,9,35], we classified AMD according to the recently published Beckmann classification system. [3] Some studies did not discriminate AMD stages at all. [34] This somewhat limits comparability between studies. However, there is growing evidence that classification into early stages (comprising early and intermediate AMD according to the Beckmann classification) and late AMD is very comparable across different AMD classification systems. [31,38,39]

Furthermore, the assessments of visual impairment differed substantially between studies with most studies assessing only HLVA. For instance, participants of the *National Health and Nutrition Examination Surveys* were categorized based on HLVA measured with an autorefractor regardless of the underlying disease.[26] As a consequence, these and other studies did not consider comparable outcomes in HLVA or other functional tests as was done in our study and might have missed associations between specific visual impairment under low luminance or low contrast situations, PA and AMD. Given our results, however, any study assessing these associations should quantify both visual impairment and PA objectively.

We found, PA to be lower with increasing age and/or presence of late AMD. Other studies using ACCs also identified visual impairment as the main factor for reduced MVPA in late AMD. [9,34] We did not find performance on any of the employed visual function tests to be associated with MVPA when including AMD stage in the model, which might be due to a difference in visual function between the early and late stage participants. Assessing the association of visual function and MVPA irrespective of AMD stage, we found LLVA to be associated. This is of particular interest as function under low luminance seems to be first and foremost affected in early AMD stages, as is reflected in our sample as well. [40,41] This finding–the association of any visual impairment with PA—is in keeping with the aforementioned studies also employing ACCs. [9,26,34]

Strengths of our study included the use of a three-dimensional waterproof ACC, which allows for a continuous measurement of PA at all times and during all conditions. Assessing PA by ACC can lead to increased levels of PA because of the constant “monitoring” of participants.[42] However, this is unlikely to affect recorded levels of PA more than the very common over-reporting of PA when using questionnaires. Further strengths of our study are the meticulous phenotyping and comprehensive assessment of any present visual impairment, in particular under low luminance and low contrast conditions which is highly relevant to AMD. Limitations include the cross-sectional nature of our study and the moderate sample size. However, using an ACC reduces the sample size needed compared to the less precise quantification of PA using questionnaires. Cut-offs for the duration of PA measurement are somewhat arbitrary and might lead to some PA relevant to the research question not being captured. However, we demonstrated that capturing PA over 30 versus 7 days did not yield any more useful data in relation to average weekly PA in a comparable group of older persons with eye diseases, which is why we chose to capture PA over 7 days in this study. [28] This of course does not account for seasonal, event-related or weather-related variations in PA. Participants were instructed to switch the ACCs on if they had a “normal” week ahead of them, without any variation to their normal PA routine which might have led to a measurement approximating the true mean of their weekly PA somewhat better. However, we have no means of verifying this. As we did not include an age-matched control group without AMD, our results cannot be transferred to a general older population. AMD patients experiencing impairment in central visual function may adapt to vision loss and compensate by using e.g. eccentric fixation. We did not capture adaptation to vision loss other than through participants’ performance in tests of central visual function. Whether adaptation to vision loss is relevant for individual PA levels over and above this needs to be assessed by future studies. We did not find lifestyle factors such as smoking to be associated with MVPA in our sample which is likely due to only a small number of smokers. Similarly, we were not able to assess the impact of concurrent other disabilities on MVPA as only two participants reported any additional disability not related to vision.

The lack of association found once we controlled for AMD stage might be explained by a clear separation into visual performance levels by AMD stage. Furthermore, the applied ACCs have not been validated against other objective measures of PA in AMD patients. Elderly, particularly visually impaired persons may have difficulties in handling the devices which may confound the measured PA. In a previous study, we evaluated the applicability of the same ACCs in an older population with eye diseases. We found a high acceptance and participants were generally able to handle the devices correctly. [28] However, the literature regarding PA measurements in older populations, in particular those with vision impairment, is sparse and no studies specifically assessing the validity of any PA measurements against performance in a lab have been published for this group.

In conclusion, we found AMD stage, but not performance in any functional test to be associated with MVPA in our cohort. Thus, the association of AMD stage and MVPA cannot entirely be explained by functional loss in late AMD. Further longitudinal research is needed to further clarify the association of PA and AMD.
